# Association between gut microbiota and chronic diseases in East Asian ethnicity: A two-sample Mendelian randomization study

**DOI:** 10.1097/MD.0000000000048291

**Published:** 2026-04-10

**Authors:** Hao Hu, Feng Xie, Ya Song, Shirui Yu, Xudong Liu

**Affiliations:** a School of Food Engineering, Moutai Institute, Zunyi, Guizhou, China; b Guizhou Health Wine Brewing Technology Engineering Research Center, Moutai Institute, Zunyi, Guizhou, China.

**Keywords:** chronic diseases, East Asian ethnicity, gut microbiota, Mendelian randomization

## Abstract

The etiology and pathogenesis of chronic diseases (CDs) are complex and may result in a variety of symptoms that negatively affect patients’ daily lives. There is growing evidence that changes in the composition and function of the gut microbiota (GM) are linked to the onset and progression of CDs, but a causal relationship between them has not yet been demonstrated in the East Asian ethnicity. We sourced data from publicly available genome-wide association study summary statistics that focused on the composition of the GM and its association with 6 specific CDs: chronic obstructive pulmonary disease (COPD), Graves disease (GD), interstitial lung disease (ILD), periodontal disease (PD), peripheral arterial disease (PAD), and coronary artery disease (CAD). We then investigated the causal relationship between the GM and the 6 CDs using a two-sample Mendelian randomization approach. To assess these causal links, we applied multiple statistical methods, including MR-Egger regression, weighted median estimation, inverse-variance weighted analysis, as well as simple and weighted mode-based approaches. Among these, the inverse-variance weighted method was selected as the primary analytical strategy. To ensure the credibility and stability of our findings, we further conducted a comprehensive set of sensitivity analyses. We found that the genus *Allisonella* significantly increased the risk of COPD, whereas LachnospiraceaeNK4A136group decreased the risk of COPD; *Anaerotruncus* increased the risk of GD, whereas *Lachnospiraceae* UCG004 decreased the risk of GD; *Catenibacterium* and *Bifidobacterium* increased the risk of ILD, while *Butyricicoccus* decreased the risk of ILD; *Catenibacterium* and *Olsenella* increased the risk of PD, whereas *Anaerostipes*, LachnospiraceaeGroupNK4A136, *Hungatella*, and *Lactococcus* reduced the risk of PD; *Parasutterella*, *CandidatusSoleaferrea*, and *Desulfovibrio* increased the risk of PAD and *Paraprevotella* reduced the risk of PAD; *Allisonella* increased the risk of CAD and *Senegalimassilia* decreased the risk of CAD. No significant heterogeneity or polynomial estimates were found. Our research indicates a direct causal link between 19 specific types of GM and 6 chronic illnesses within East Asian populations. This contributes fresh understanding of how the composition of GM influences the development and progression of CDs.

## 1. Introduction

Recent studies have delved deeply into the significance of the gut microbiota (GM) in influencing both human health and various disease states, highlighting the intricate interplay among the gut microbial community, dietary patterns, lifestyle behaviors, and inherited genetic traits.^[1–3]^ Of particular relevance is the emerging evidence suggesting that the composition and function of the GM may play a crucial role in the initiation and advancement of chronic illnesses. Mendelian randomization (MR), a statistical analytical approach that leverages genetic variations to serve as indirect indicators of changeable risk factors, has gained prominence as an effective methodology for examining cause-and-effect relationships between the GM and the development of chronic diseases (CDs).^[4,5]^

CDs are long-lasting, theoretically incurable, and insoluble, and account for a significant portion of the medical concerns affecting older adults.^[6]^ The 2017 Report on Global Health Statistics published by the World Health Organization highlights that CDs are responsible for more than 41 million deaths globally, accounting for 71.3% of all deaths. Various studies have shown that CDs, such as chronic obstructive pulmonary disease (COPD),^[7]^ Graves disease (GD),^[8]^ interstitial lung disease (ILD),^[9]^ periodontal disease (PD),^[10]^ peripheral arterial disease (PAD),^[11]^ and coronary artery disease (CAD),^[12]^ are associated with alterations in the GM. However, determining a clear cause-and-effect relationship between the GM and these diseases is challenging due to potential confounding factors. MR, which utilizes genetic variations that are linked to the composition of the GM as an unbiased proxy for exposure to the GM, offers a unique opportunity to address these challenges.

In this paper, we aim to use MR to explore the potential impact of GM on the occurrence and progression of COPD, GD, ILD, PD, PAD, and CAD. We will examine the relevant literature, highlight key studies that have applied MR to examine these associations, and discuss the strengths and limitations of this method of analysis. Our goal is to provide a comprehensive synthesis of the current evidence on the role of the GM in these CDs and to identify potential avenues for future research.

## 2. Methods

### 2.1. Study design

**Figure 1.**
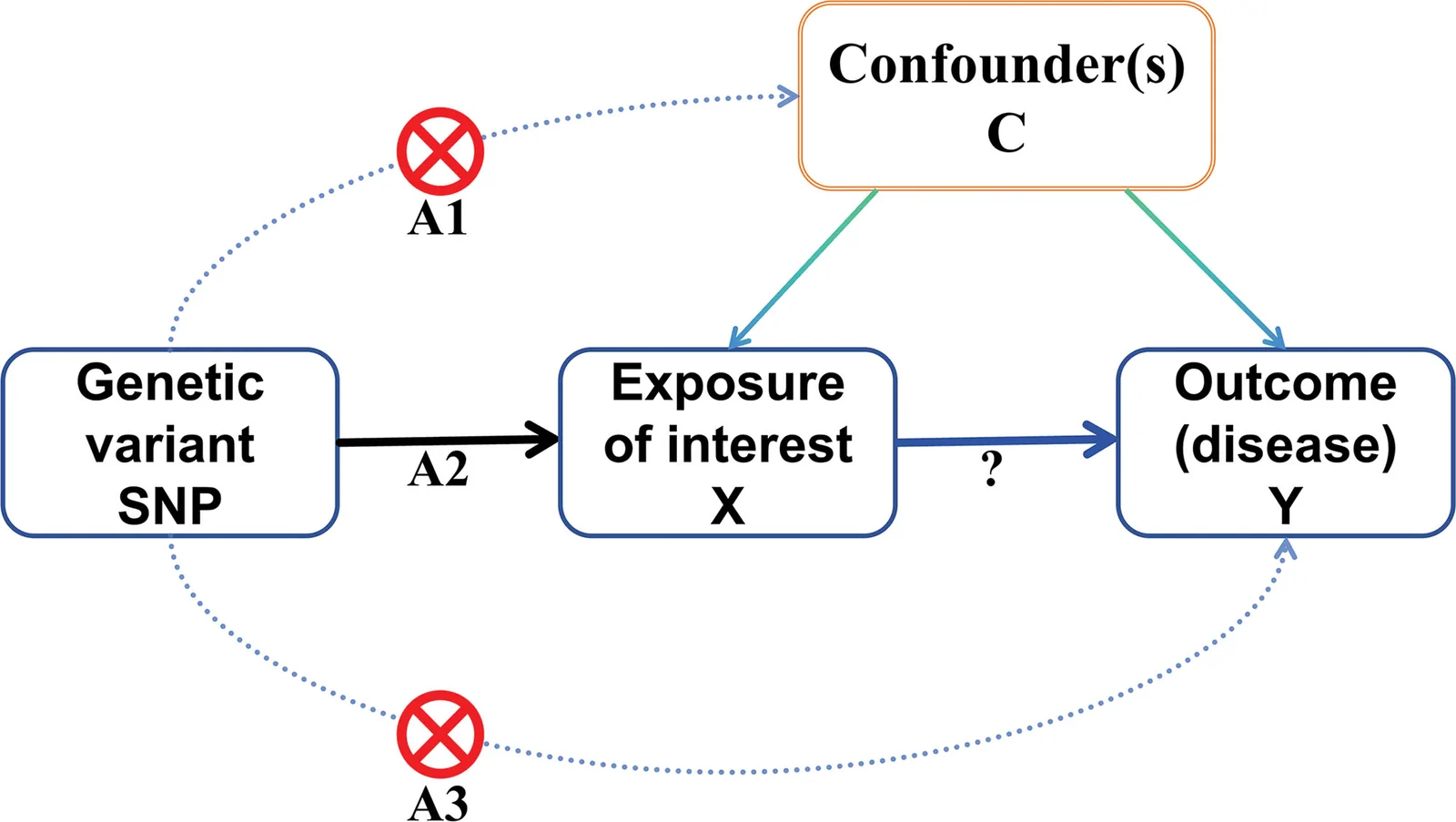
The illustrative diagram of two-sample Mendelian randomization analysis. A1: No unmeasured confounders of the association between SNP and C. A2: SNP must be associated with X. A3: SNP must only affect health and disease through X, and not Y. SNP = single-nucleotide polymorphism.

In this investigation, we leveraged publicly accessible data derived from the genome-wide association study (GWAS) abstracts to select suitable instrumental variables (IVs) for MR analyses. The primary objective was to explore the potential causal links between GM composition and 6 specific CDs within the East Asian population. To maintain the reliability and validity of our findings, we rigorously followed 3 key assumptions inherent to MR methodology: first, the selected IVs must demonstrate a robust association with the exposure of interest (i.e., GM); second, the IVs should not exhibit any correlation with potential confounding variables that could bias the results; and third, the influence of the IVs on the outcome should be mediated solely through the exposure, thereby ensuring a clear causal pathway (Fig. 1). It is important to note that the original study from which the data were obtained had already secured the necessary ethical approvals and informed consent from participants.

### 2.2. Data sources

This investigation is a secondary analysis of GWAS data that are available to the public, with ethical approval granted for all original data. Genetic information related to the gut microbiome for genera is derived from the latest GWAS data from the MiBioGen database, which integrates whole-genome genotyping information from 24 independent cohorts totaling 18,340 individuals, along with fecal microbial community composition data obtained through 16S rRNA sequencing technology. GWAS summary data for COPD, GD, ILD, PD, PAD, and CAD were obtained from the IEU Open GWAS database (https://gwas.mrcieu.ac.uk/). The database contains 346,601,917,583 genetic associations from 50,044 GWAS summary datasets. Participants were recruited by 44 institutions/consortia and diagnosed according to established consensus criteria. Microbiome GWAS was adjusted for age, sex, technical covariates, and genetic principal components. Cutoff thresholds included: minor allele frequency >0.05 and single-nucleotide polymorphism (SNP)-wise call filtering >0.95. To ensure consistency of the study, all outcome GWAS data used were from East Asian populations. All GWAS data used in the analyses are detailed in Table S1, Supplemental Digital Content, https://links.lww.com/MD/R635.

### 2.3. IVs selection

**Figure 2.**
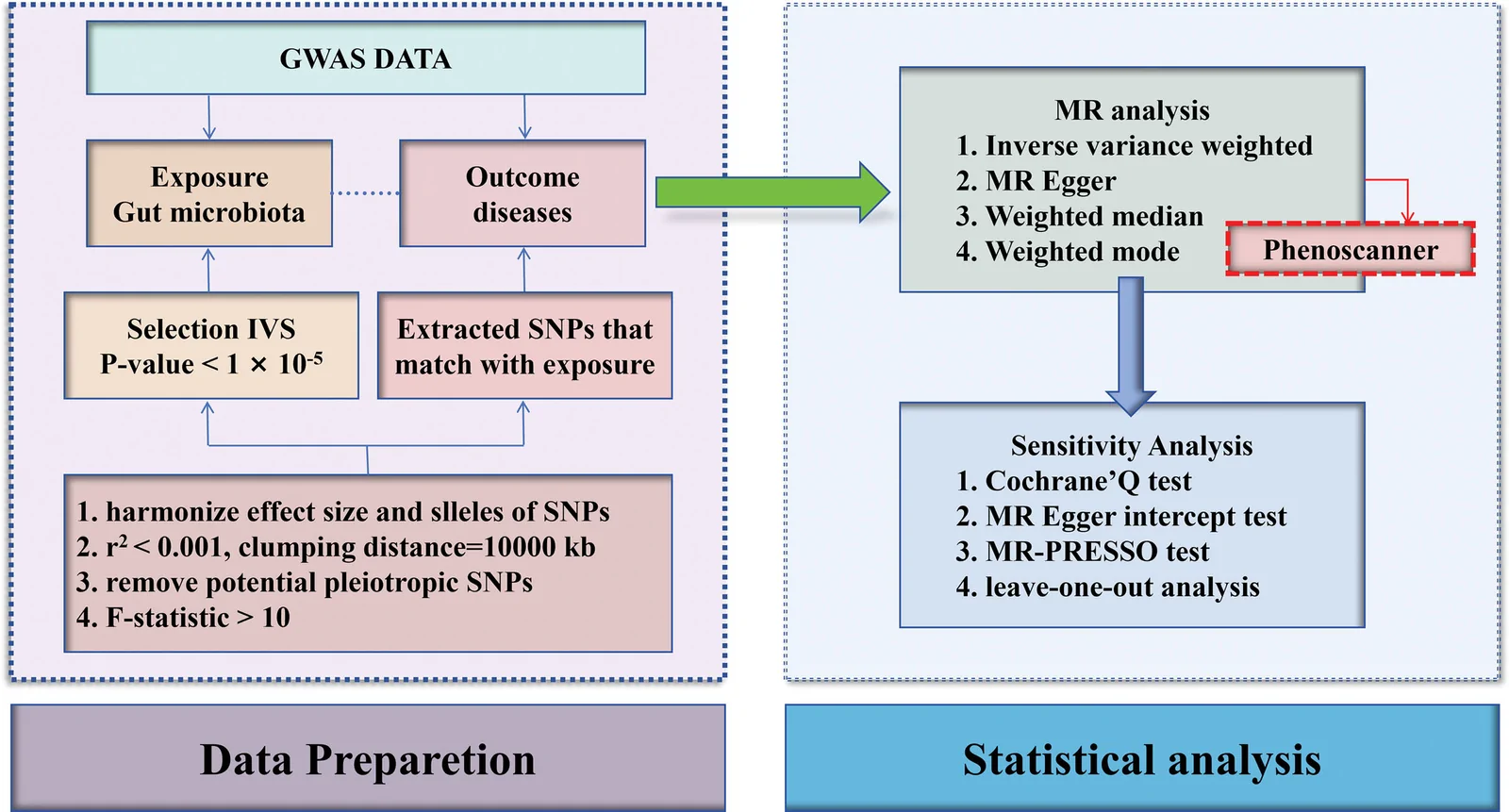
The whole workflow of MR analysis. GWAS = genome-wide association study, IVs = instrumental variables, MR = Mendelian randomization, SNP = single-nucleotide polymorphism.

Data from 131 taxa were processed (Fig. 2), first, using a threshold of *P* < 1 × 10^−8^, as the number of SNPs for which genome-wide significance could be obtained is limited and this loose threshold has been used in many studies^[13]^; second, a linkage disequilibrium test was carried out to exclude *r*^2^ < 0.001 within the 10,000 kb window^[14]^; and third, the *F*-statistic for each SNP was calculated to exclude SNPs with *F* < 10 to avoid weak instrumental bias. After rigorous screening, the identified IVs and corresponding *F*-statistics are shown in Table S2, Supplemental Digital Content, https://links.lww.com/MD/R635.

### 2.4. MR analysis

To assess the causal relationship between GM and disease, we conducted independent two-sample MR analyses. The primary analytical approach utilized was the inverse-variance weighted (IVW) method, while the Wald ratio test was applied specifically for single IV scenarios. The outcomes of the MR analyses were reported as odds ratios (ORs) along with their associated 95% confidence intervals (CIs). Statistical significance was determined based on an IVW *P*-value threshold of <.05.

### 2.5. Sensitivity analysis

We conducted Cochran *Q* tests to evaluate the degree of heterogeneity among the individual SNPs and created scatter plots illustrating the correlations between each SNP and both the exposure and the outcome variables, thereby providing a visual representation of the MR findings. To investigate the influence of each SNP on the overall results, we carried out leave-one-out analyses, systematically excluding 1 SNP at a time and then rerunning IVW MR analyses on the remaining SNPs to determine whether any specific genetic variant might be driving the observed associations. Furthermore, we employed Mendelian randomization pleiotropy residual sum and outlier (MR-PRESSO) and MR-Egger regression methods to screen for potential horizontal pleiotropic effects. Specifically, MR-PRESSO was utilized to identify statistically significant outliers and to adjust for horizontal pleiotropy by excluding those outlier SNPs from the analysis. All statistical analyses were executed using the R programming language (version 4.4.0), and the MR analyses were carried out with the R package “Two Sample MR.”

## 3. Results

### 3.1. IV selection

For the 131 taxa we studied, we provide the results of 1531 SNP identification for each microbial taxon (Table S2, Supplemental Digital Content, https://links.lww.com/MD/R635). The detailed data for each SNP encompasses the effect allele, the other allele, the effect size (β), the standard error, the *P*-value (pval), the coefficient of determination (*R*^2^), and the *F*-statistic (*F*-value). A search of the SNPs using PhenoScanner did not identify any associations with the specified potential confounding variables.

### 3.2. Causal effects of GM on multiple 6 diseases

#### 3.2.1. COPD

**Figure 3.**
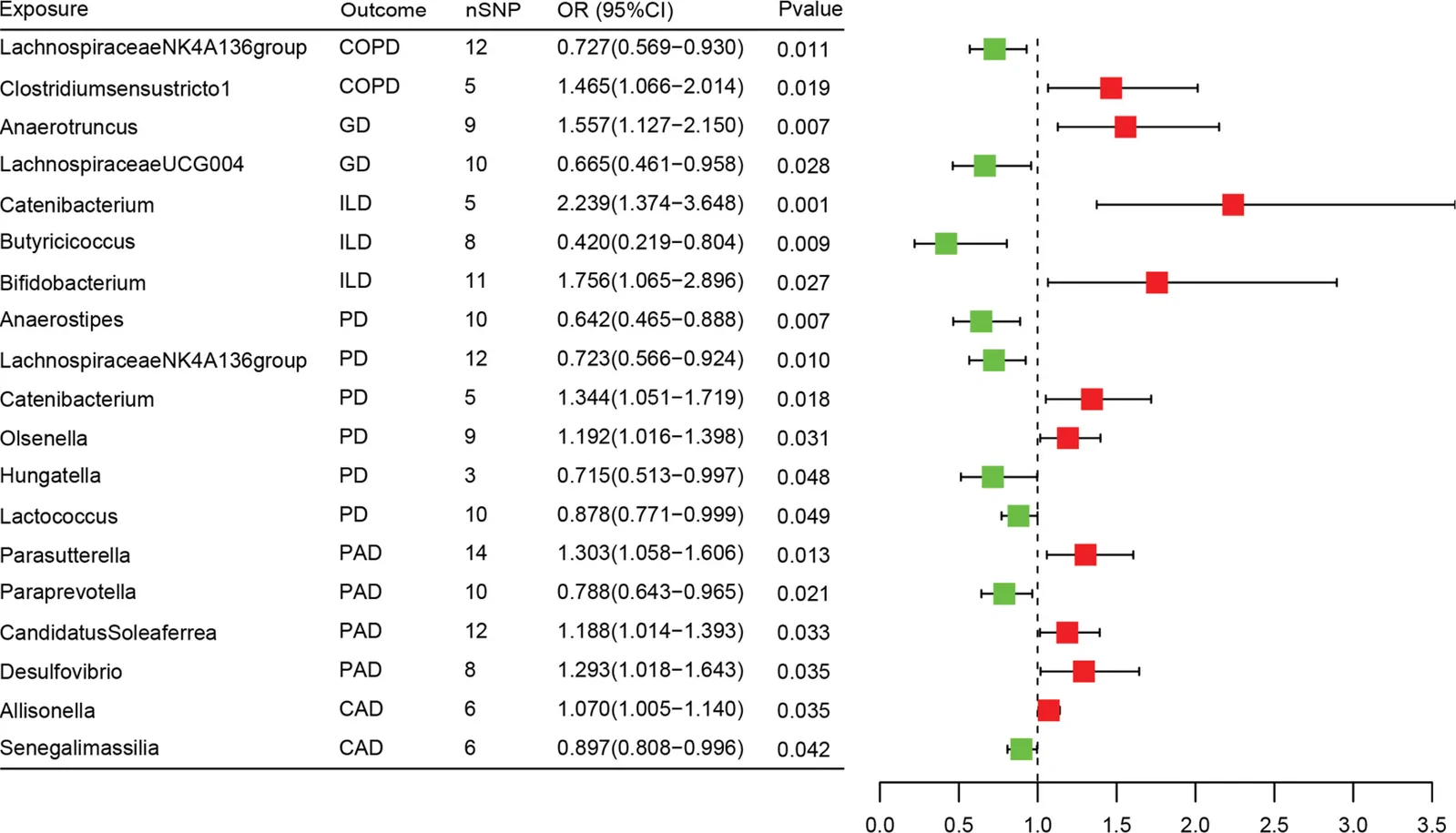
Forest plot of Mendelian randomization estimates between gut microbiota and chronic diseases. The figure showed the IVW estimates of significant chronic diseases associated with gut microbiota taxa. The blocks represent the IVW estimates, and the black bars represent the 95% confidence intervals of the IVW estimates. The OR > 1 (red blocks) indicates increased risk, while OR < 1 (green blocks) indicates decreased risk. CAD = coronary artery disease, CI = confidence interval, COPD = chronic obstructive pulmonary disease, GD = Graves disease, ILD = interstitial lung disease, IVW = inverse-variance weighted, OR = odds ratio, PAD = peripheral arterial disease, PD = periodontal disease, SNP = single-nucleotide polymorphism.

A total of 2 GMs (including Clostridiumsensustricto1 and LachnospiraceaeNK4A136group) were associated with COPD (Table S3, Supplemental Digital Content, https://links.lww.com/MD/R635, Fig. 3). As shown in Figure 3, the genus Clostridiumsensustricto1 (OR = 1.465, 95% CI = 1.066–2.014, *P* = .019) significantly increased the risk of COPD, whereas the genus LachnospiraceaeNK4A136group (OR = 0.727, 95% CI = 0.569–0.930, *P* = .011) significantly decreased the risk of COPD.

#### 3.2.2. GD

Two GMs (including *Anaerotruncus* and *Lachnospiraceae* UCG004) were associated with GD (Table S3, Supplemental Digital Content, https://links.lww.com/MD/R635, Fig. 3). The genus *Anaerotruncus* (OR = 1.557, 95% CI = 1.127–2.150, *P* = .007) is found to be positively associated with GD. The genus *Lachnospiraceae* UCG004 (OR = 0.665, 95% CI = 0.461–0.958, *P* = .028) is found to be negatively associated with GD.

#### 3.2.3. ILD

A total of 3 GMs, including *Catenibacterium*, *Bifidobacterium*, and *Butyricicoccus*, were associated with ILD (Table S3, Supplemental Digital Content, https://links.lww.com/MD/R635, Fig. 3). The genus *Catenibacterium* and *Bifidobacterium* are found to be positively associated with ILD, suggesting that genus *Catenibacterium* (OR = 2.239, 95% CI = 1.374–3.648, *P* = .001) and *Bifidobacterium* (OR = 1.756, 95% CI = 1.065–2.896, *P* = .027) were causally related to an increased risk of ILD. The genus *Butyricicoccus* (OR = 0.420, 95% CI = 0.219–0.804, *P* = .009) is causally related to a decreased risk of ILD.

#### 3.2.4. PD

A total of 6 GMs, including *Anaerostipes*, LachnospiraceaeNK4A136group, *Catenibacterium*, *Olsenella*, *Hungatella*, and *Lactococcus*, were associated with PD (Table S3, Supplemental Digital Content, https://links.lww.com/MD/R635, Fig. 3). The genus *Catenibacterium* and *Olsenella* were found to be positively associated with PD, suggesting that genus *Catenibacterium* (OR = 1.344, 95% CI = 1.051–1.719, *P* = .018) and *Olsenella* (OR = 1.192, 95% CI = 1.016–1.398, *P* = .031) were causally related to an increased risk of PD. The genus *Anaerostipes*, LachnospiraceaeNK4A136group, *Hungatella*, and *Lactococcus* were found to be negatively associated with PD, suggesting that genus *Anaerostipes* (OR = 0.642, 95% CI = 0.465–0.888, *P* = .007), LachnospiraceaeNK4A136group (OR = 0.723, 95% CI = 0.566–0.924, *P* = .010), *Hungatella* (OR = 0.715, 95% CI = 0.513–0.997, *P* = .048), and *Lactococcus* (OR = 0.887, 95% CI = 0.771–0.999, *P* = .049) were causally related to a decreased risk of PD.

#### 3.2.5. PAD

A total of 4 GMs, including *Parasutterella*, *Paraprevotella*, *CandidatusSoleaferrea*, and *Desulfovibrio*, were associated with PAD (Table S3, Supplemental Digital Content, https://links.lww.com/MD/R635, Fig. 3). The genus *Parasutterella*, *CandidatusSoleaferrea*, and *Desulfovibrio* were found to be positively associated with PAD, suggesting that genus *Parasutterella* (OR = 1.303, 95% CI = 1.058–1.606, *P* = .013), *CandidatusSoleaferrea* (OR = 1.188, 95% CI = 1.014–1.393, *P* = .033), and *Desulfovibrio* (OR = 1.293, 95% CI = 1.018–1.643, *P* = .035) were causally related to an increased risk of PAD. The genus *Paraprevotella* (OR = 0.788, 95% CI = 0.643–0.965, *P* = .021) is causally related to a decreased risk of PAD.

#### 3.2.6. CAD

Two GMs (including *Allisonella* and *Senegalimassilia*) were associated with CAD (Table S3, Supplemental Digital Content, https://links.lww.com/MD/R635, Fig. 3). The genus *Allisonella* is found to be positively associated with CAD, suggesting that genus *Allisonella* (OR = 1.070, 95% CI = 1.005–1.140, *P* = .035) is causally related to an increased risk of CAD. The genus *Senegalimassilia* is found to be negatively associated with CAD, suggesting that genus *Senegalimassilia* (OR = 0.897, 95% CI = 0.808–0.996, *P* = .042) is causally related to a decreased risk of CAD.

### 3.3. Sensitivity analyses

We employed the Cochran *Q* test to assess heterogeneity and utilized the MR-PRESSO test and MR-Egger intercept analysis to identify horizontal pleiotropy. Test results indicated no evidence of heterogeneity or horizontal pleiotropy (*P* > .05) (Table S4, Supplemental Digital Content, https://links.lww.com/MD/R635). Leave-one-out analysis further confirmed the reliability of the MR analysis (Figures S1–S6, Supplemental Digital Content, https://links.lww.com/MD/R636). Scatter plots illustrated the overall effects of GM on 6 CDs (Figures S7–S12, Supplemental Digital Content, https://links.lww.com/MD/R636). Additionally, forest plots revealed causal associations between GM and these 6 CDs (Figures S13–S18, Supplemental Digital Content, https://links.lww.com/MD/R636).

## 4. Discussion

In our study, a two-sample MR analysis effectively identified that specific components of the GM may either contribute to the development of or offer protection against CDs. The findings underwent rigorous evaluation through multiple analytical methods, including the IVW approach, MR-Egger regression, weighted median estimation, as well as simple and weighted mode-based analyses. The results from nearly all these methods demonstrate a high degree of consistency in revealing potential causal relationships. We analyzed the relationships between 131 common GM abundances and 6 types of CDs (COPD, GD, ILD, PD, PAD, and CAD). The results showed that some GMs were risk factors, and some were protective factors for each chronic disease. This is the first comprehensive assessment of the potential relationship between GM and CDs in East Asian ethnicity. However, additional research is required to elucidate the influence of GM on CDs and to uncover the exact biological processes involved.

Numerous observational research studies have documented a correlation between the composition and diversity of GM and the development or progression of various chronic illnesses. In our research results, there are 19 GMs associated with diseases. *Clostridium butyricum* and its derived extracellular vesicles can regulate intestinal homeostasis and improve acute experimental colitis.^[15]^ Due to the reduction of intestinal pathogens, such as *Escherichia coli, Shigella*, and Clostridiumstrictum1, symptoms such as diarrhea, rectal bleeding, colonic mucosal damage, congestion, and edema have improved.^[16]^ The treatment with *C butyricum* has been shown to improve the immunotherapeutic effect of lung cancer,^[17]^ which is consistent with our research findings. The Lachnospiraceae family of bacteria residing in the organization promotes tumor immune surveillance to prevent the occurrence of colorectal cancer.^[18]^ Several microbial species, such as Streptococcus sp000187445 and *Streptococcus vestibularis*, along with various organisms belonging to the Lachnospiraceae family, have been linked to a reduction in pulmonary function,^[18]^ further confirming our research findings. An increase in anaerobic bacteria has been confirmed in human patients with hypercholesterolemia.^[19]^ There are few research reports on anaerobic bacteria and GD. Fecal samples were collected from 55 GD patients and 48 healthy controls, and linear discriminant analysis showed an increase in Lachnospiraceae in healthy controls.^[20]^ The decrease in the probiotic *Catenibacterium* studied by Quan et al may be related to pulmonary fibrosis,^[21]^ whereas we investigated that *Catenibacterium* increases the risk of ILD. *C butyricum* exerts its antitumor effect by generating butyric acid, which is consistent with our research findings.^[22]^ Man et al investigated the relationship between 6 microorganisms (*Gemella*, *Actinobacteria*, *Prevotella*, *Neisseria*, *Haemophilus*, and *Bifidobacterium*) and ILD^[23]^ but did not clarify the correlation between *Bifidobacterium* and ILD. Currently, there is limited research exploring the relationship between anaerobic bacteria and PD. Among the subgingival microbial communities in the study subjects, a relatively high average detection rate has been observed for several microorganisms, including *Enterobacterium*, *Peptostreptococcus anaerobius*, *Candida albicans*, *Pseudomonas aeruginosa*, *Eubacterium saphenum*, *Clostridium difficile*, and *Olsenella uli*.^[24]^ When observing the subgingival pocket and tumor tissue of CRC patients, a symbiotic pattern of these oral microbiota was found, which is also associated with other intestinal microbiota, such as *Hungatella*.^[25]^ Wuttke et al achieved good results in the treatment of periodontitis using *Lactococcus*.^[26]^ Hoffman et al found a significant difference in the expression of *Parasutterella* between patients with and without arteritis.^[27]^ Zhao et al found a negative correlation between *Paraprevotella* and cardiovascular disease.^[28]^ Although there have been studies on the association between *Candidatus Soleaferrea* and various diseases, there have been no reports on PADs. A Japanese man developed an aneurysm infection, and clinical doctors should suspect *Desulfovibrio* infection in similar situations.^[29]^ Our results confirm this hypothesis. *Allisonella*’s research results are consistent with literature reports.^[30]^ Due to the low detection rate of *Senegalimassilia*, its association with arteries was excluded,^[31]^ and our research results further enhance this conclusion.

In this paper, we examine these CDs and their potential relationships with GM. COPD represents a significant global health burden, being one of the leading contributors to both illness and death around the world. This progressive respiratory condition primarily includes 2 main pathological entities: chronic bronchitis, which is characterized by persistent cough and mucus production, and emphysema, which involves damage to the air sacs (alveoli) in the lungs, leading to breathing difficulties. The exact cause is not yet clear, and it may be the result of the combined action of internal factors (individual susceptibility factors) and external factors (environmental factors). In most patients, COPD is often combined with other CDs with obvious clinical symptoms, which will increase the incidence rate and mortality of COPD. It has been demonstrated that both individuals with COPD and smokers who have preserved normal lung function can experience vascular wall remodeling and the onset of pulmonary hypertension. This finding implies that structural changes in the blood vessels may play a contributing role in the pathogenesis of emphysema.^[32]^ The GM is another reason for the development of emphysema. Notably, the levels of beneficial bacteria, such as *Lactobacillus* and *Akkermansia*, were reduced, while the levels of potentially pathogenic bacteria, such as Proteobacteria, were increased in COPD patients. We have found a correlation between 2 types of GM and COPD. The LachnospiraceaeNK4A136group is beneficial and *Clostridium* sensu stricto 1 is harmful. GD, also known as diffuse thyroid enlargement, is a prevalent endocrine disorder. Although the precise etiology of diffuse goiter remains elusive, emerging research suggests a potential role of GM. For instance, a study by Li et al demonstrated that patients with diffuse goiter exhibited altered GM composition compared with healthy controls.^[33]^ ILD refers to a heterogeneous group of disorders affecting the lung interstitium. Although the pathogenesis of ILD is complex and not completely understood, recent evidence suggests a potential role of GM. A study by Manichaikul et al investigated the GM profiles of patients with idiopathic pulmonary fibrosis, a form of ILD, and identified significant differences compared with healthy controls.^[34]^ PDs are oral inflammatory diseases affecting the tissues supporting and surrounding the teeth. The global occurrence of PDs is believed to fall within the range of 20% to 50%, making it the 11th most common health condition according to findings from the 2016 Global Burden of Disease Study. As a result, this harmful condition is now widely recognized as a significant public health concern on a global scale. Oral bacteria can cause microbial substances to enter the bloodstream and disseminate to various organs throughout the body, and PD has also been associated with chronic, low-level inflammation that affects the entire system. In individuals diagnosed with periodontitis, the relative abundance of certain bacterial phyla – specifically Firmicutes, Proteobacteria, Verrucomicrobia, and Euryarchaeota – was found to be elevated when compared with those with healthy periodontal conditions. Conversely, the abundance of the phylum Bacteroidetes was notably reduced in patients suffering from periodontitis relative to their healthy counterparts.^[35]^ The 6 GM we discovered supplemented this result. PAD is principally recognized as a clinical expression of systemic atherosclerosis, typically manifesting as a chronic condition characterized by the narrowing or blockage of arteries in the lower limbs. It is a highly prevalent disorder, impacting over 200 million people worldwide, with reported prevalence rates varying between 3% and 10% across different populations. Contrary to our research findings, the reason may be that the selected population is not consistent. CAD stands as one of the primary contributors to global mortality. Extensive research has been conducted on the involvement of the GM in the progression of atherosclerosis and acute myocardial infarction. Nevertheless, the potential connections between the composition and function of the GM and the development of CAD itself, as well as its other associated risk factors, have not yet been thoroughly investigated.^[36]^ Accumulating scientific research indicates that changes in the composition and function of the GM may contribute to the development and progression of cardiovascular diseases. We only found 2 GMs significantly associated with CAD in the East Asian population.

## 5. Conclusion

In this investigation, we conducted an in-depth and comprehensive analysis of the causal relationships between GM and CDs. Our findings identified a total of 19 causal associations, comprising 10 positive and 9 negative links, between genetically predicted GM composition and the development of various chronic conditions. This research represents the first systematic and wide-ranging evaluation aimed at uncovering the potential connections between gut microbial communities and CDs. Nevertheless, additional research is essential to elucidate the specific ways in which GM influences the onset and progression of chronic illnesses, as well as to clarify the exact underlying biological mechanisms involved.

## 6. Clinical implications

This study sought to ascertain the impact of gut microbes on CDs, categorizing them as either “helpful” or “harmful” based on their relative abundance. Nonetheless, the specific pathways through which GM contributes to the development of CDs remain elusive. Further clinical validation is required to solidify these findings for the purposes of clinical research.

## Acknowledgments

We wish to acknowledge the researchers and participants of the GWAS used in our study.

## Author contributions

**Funding acquisition:** Ya Song, Shirui Yu, Xudong Liu.

**Methodology:** Hao Hu, Feng Xie.

**Formal analysis:** Hao Hu, Ya Song.

**Validation:** Shirui Yu.

**Software:** Xudong Liu.

**Writing – review & editing:** Hao Hu, Feng Xie.

## Supplementary Material




