# Supplementary material for: Association between gut microbiota and chronic diseases in East Asian ethnicity: A two-sample Mendelian randomization study
Source: Medicine (Baltimore). 2026 Apr 10;105(15):e48291. doi: 10.1097/MD.0000000000048291 (PMC13592929; doi:10.1097/MD.0000000000048291)
Supplement: Supplementary file 1 [file medi-105-e48291-s002.pdf]

Figure S1. MR leave-one-out sensitivity analysis for Gut microbiota on COPD.

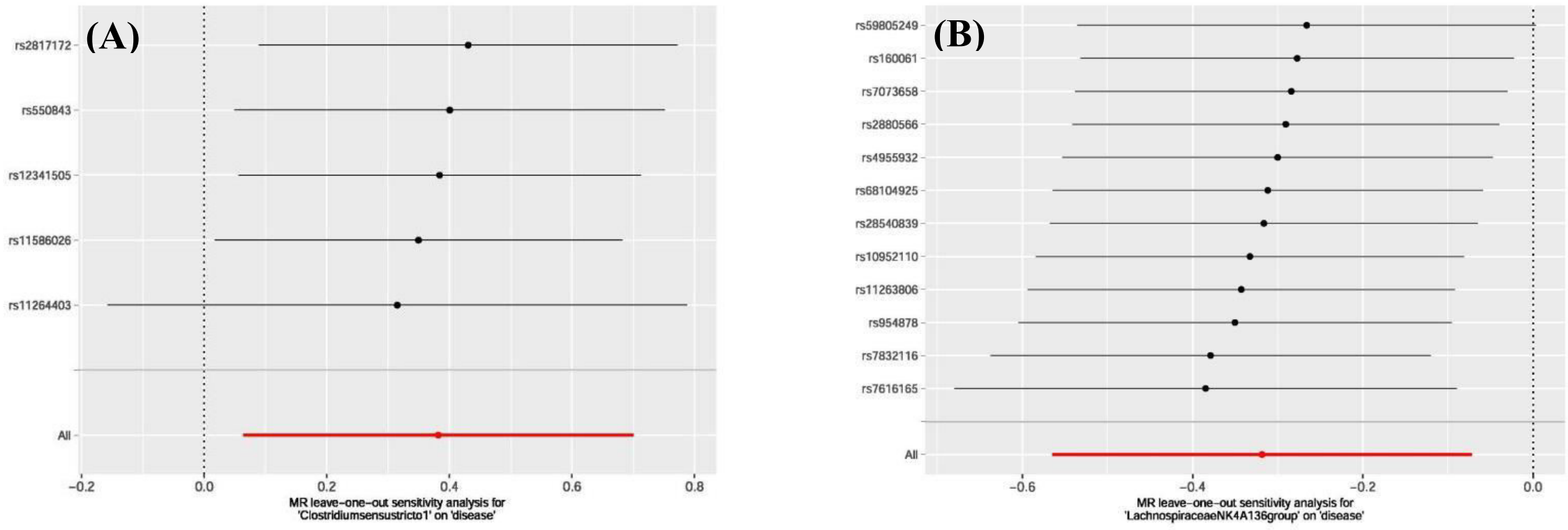

(A) Analysis for "LachnospiraceaeNK4A136group" on "COPD"

(B) Analysis for "Clostridiumsensustricto1" on "COPD"

Figure S2. MR leave-one-out sensitivity analysis for Gut microbiota on GD.

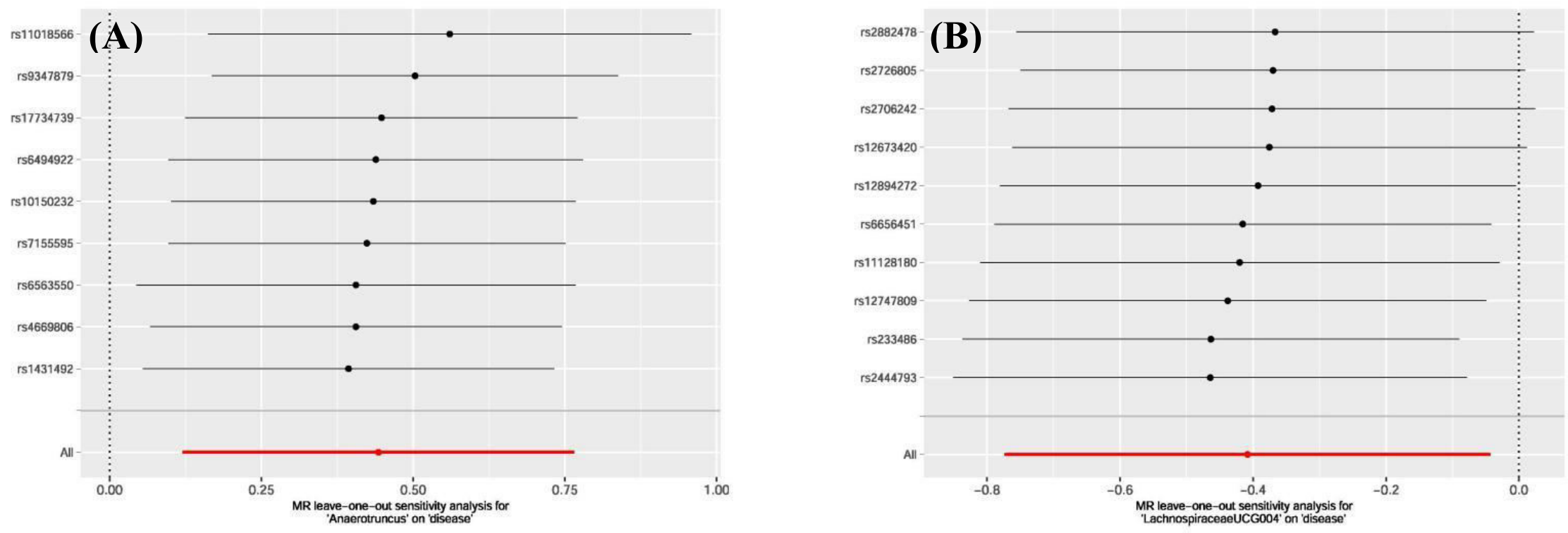

(A) Analysis for "LachnospiraceaeUCG004" on "GD"
(B) Analysis for "Anaerotruncus" on "GD"

Figure S3. MR leave-one-out sensitivity analysis for Gut microbiota on ILD.

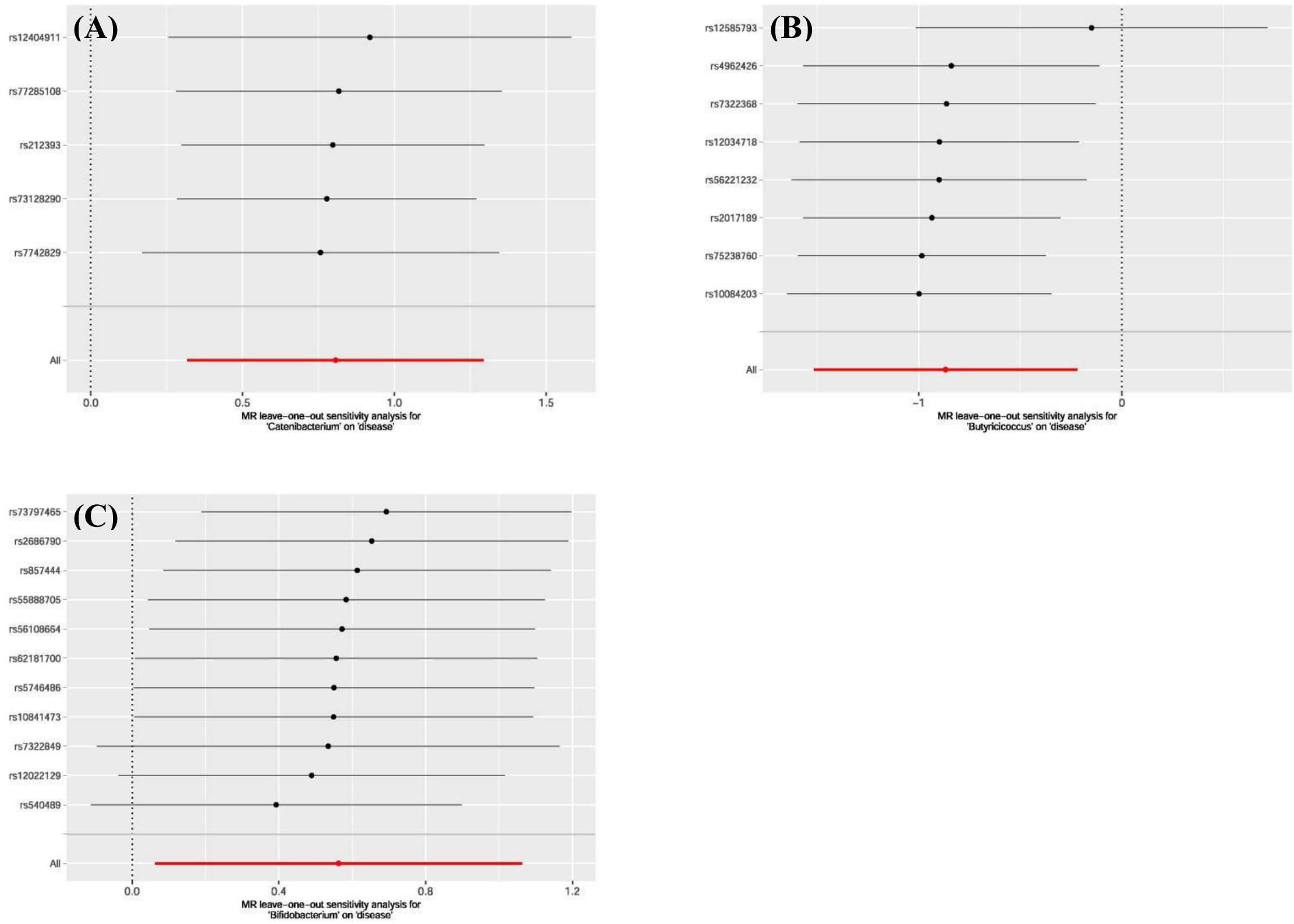

(A) Analysis for "Catenibacterium" on "ILD"
(B) Analysis for "Butyricicoccus" on "ILD"
(C) Analysis for "Bifidobacterium " on "ILD"

Figure S4. MR leave-one-out sensitivity analysis for Gut microbiota on PD.

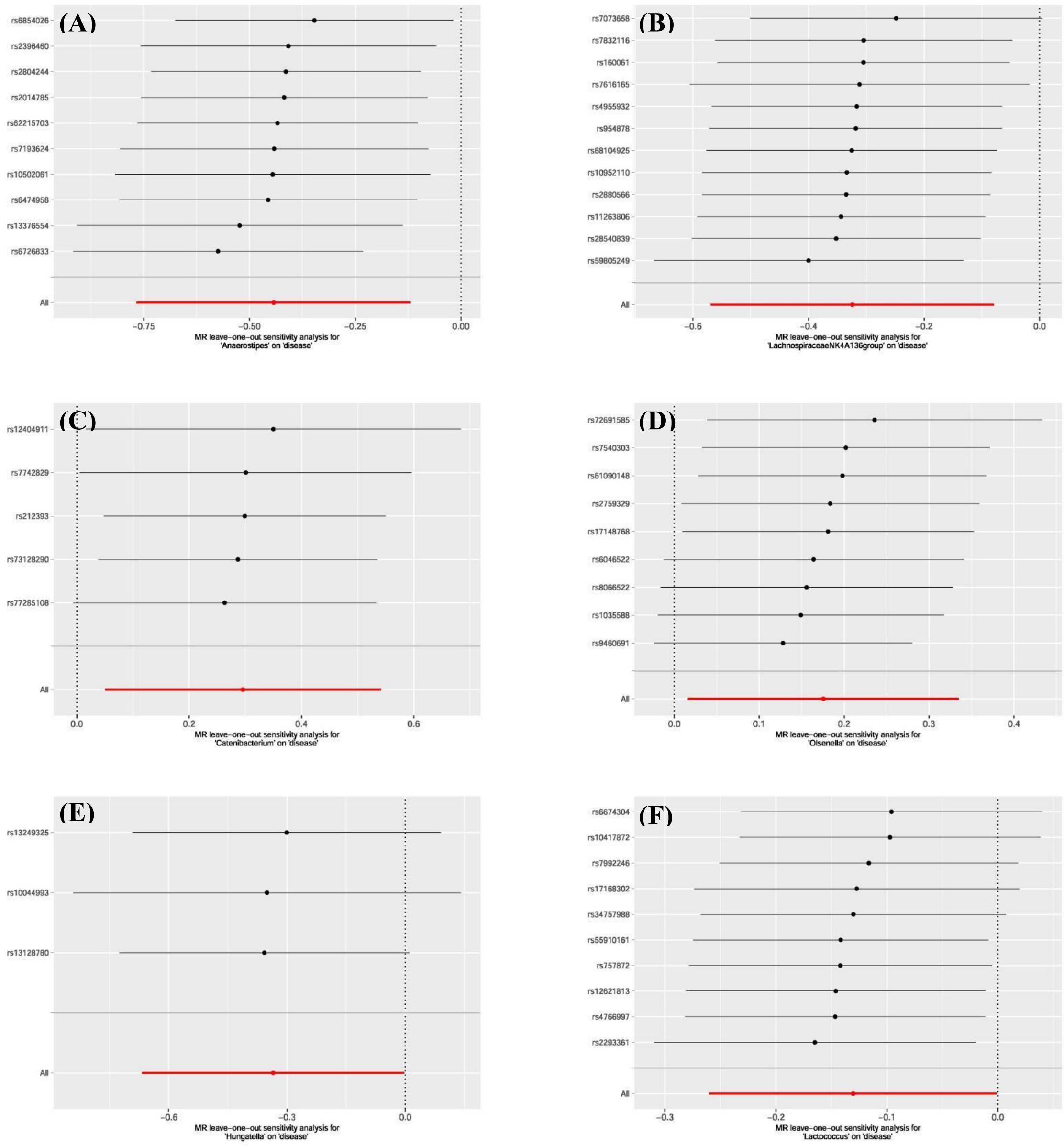

(A) Analysis for "Catenibacterium" on "PD"
(B) Analysis for "Olsenella" on "PD"
(C) Analysis for "LachnospiraceaeNK4A136group" on "PD"
(D) Analysis for "Hungatella" on "PD"
(E) Analysis for "Anaerostipes" on "PD"
(F) Analysis for "Lactococcus" on "PD"

Figure S5. MR leave-one-out sensitivity analysis for Gut microbiota on PAD.

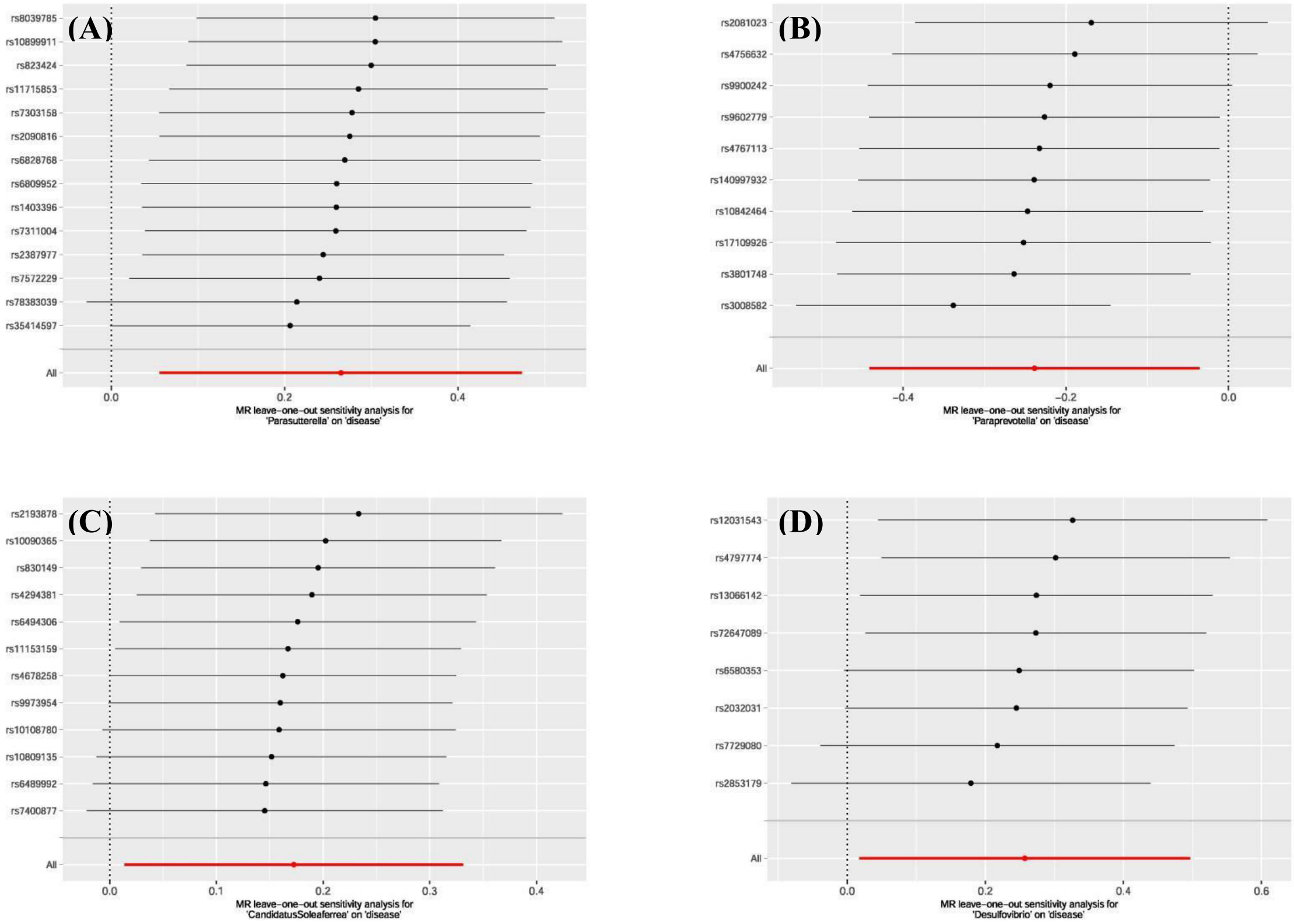

(A) Analysis for "Desulfovibrio" on "PAD"
(B) Analysis for "Paraprevotella" on "PAD"
(C) Analysis for "CandidatusSoleaferrea" on "PAD"
(D) Analysis for "Parasutterella" on "PAD"

Figure S6. MR leave-one-out sensitivity analysis for Gut microbiota on CAD.

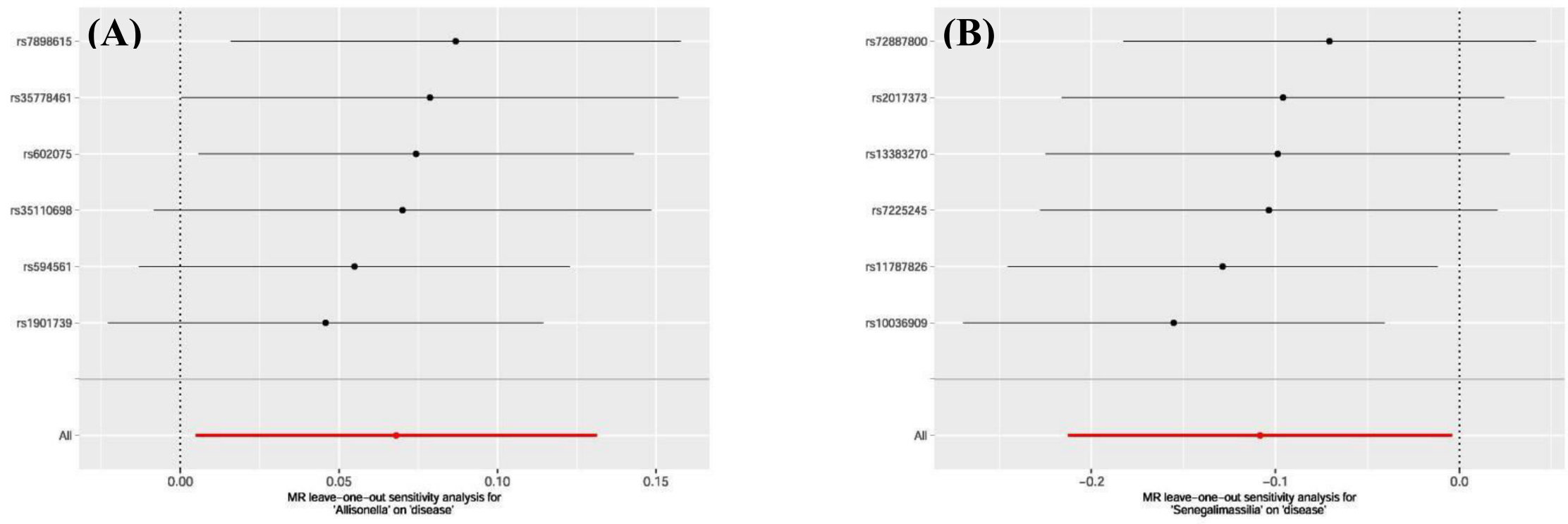

(A) Analysis for "Senegalimassilia" on "CAD"
(B) Analysis for "Allisonella " on "CAD"

Figure S7. Scatter plots for the effect of Gut microbiota on COPD.

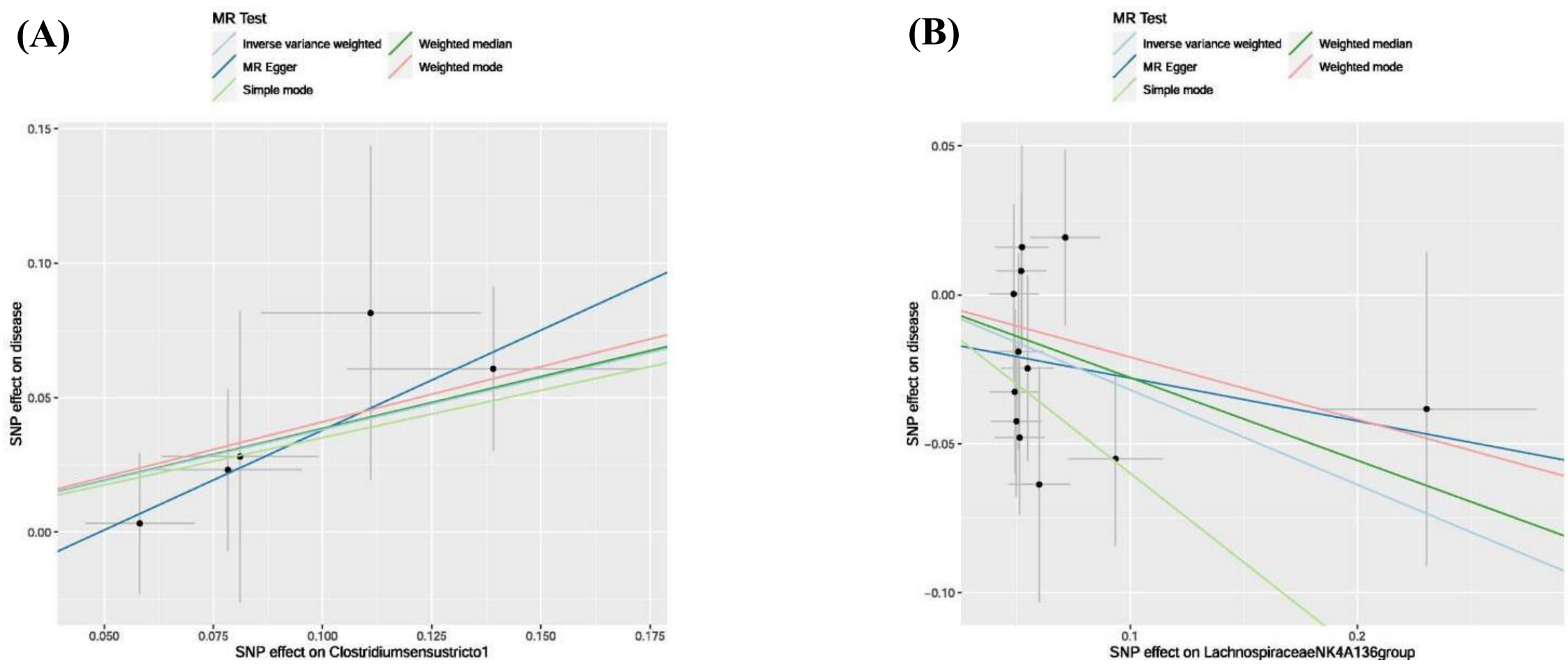

Figure S8. Scatter plots for the effect of Gut microbiota on GD.

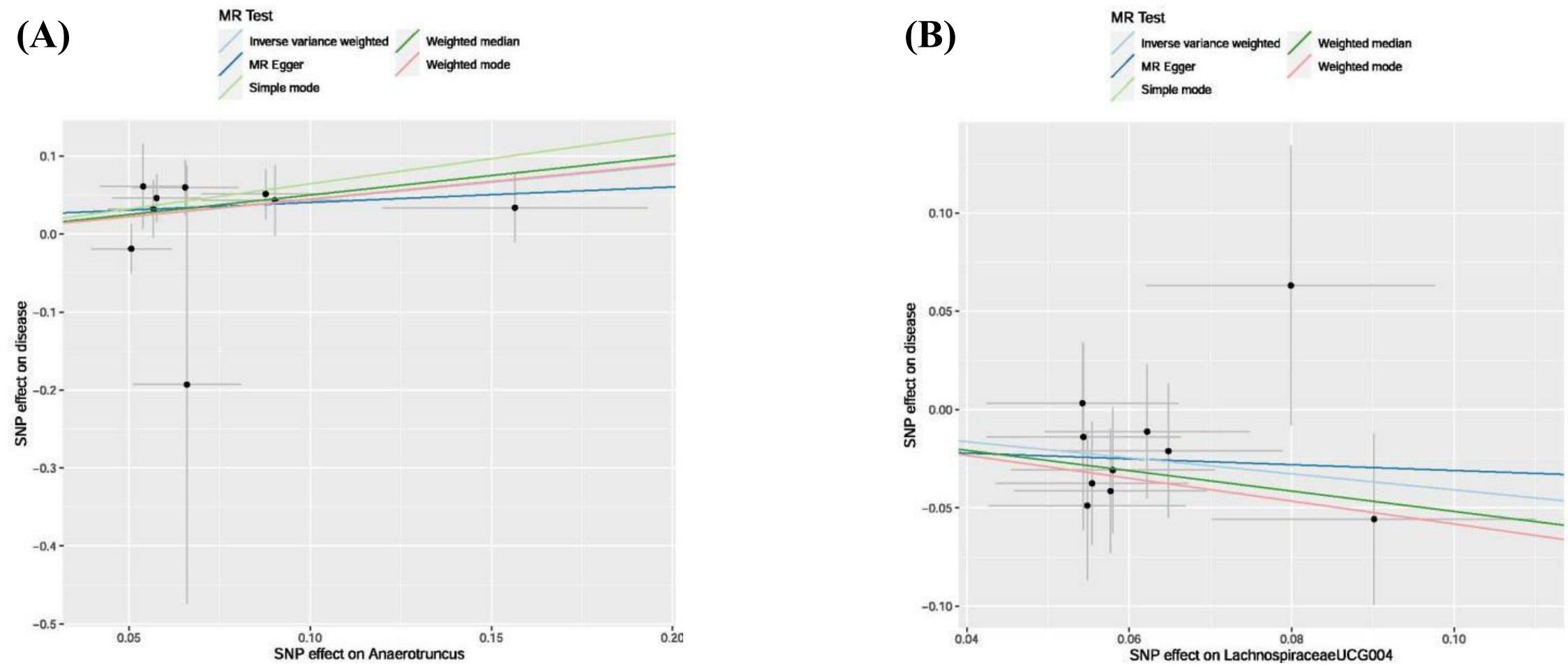

Figure S9. Scatter plots for the effect of Gut microbiota on ILD.

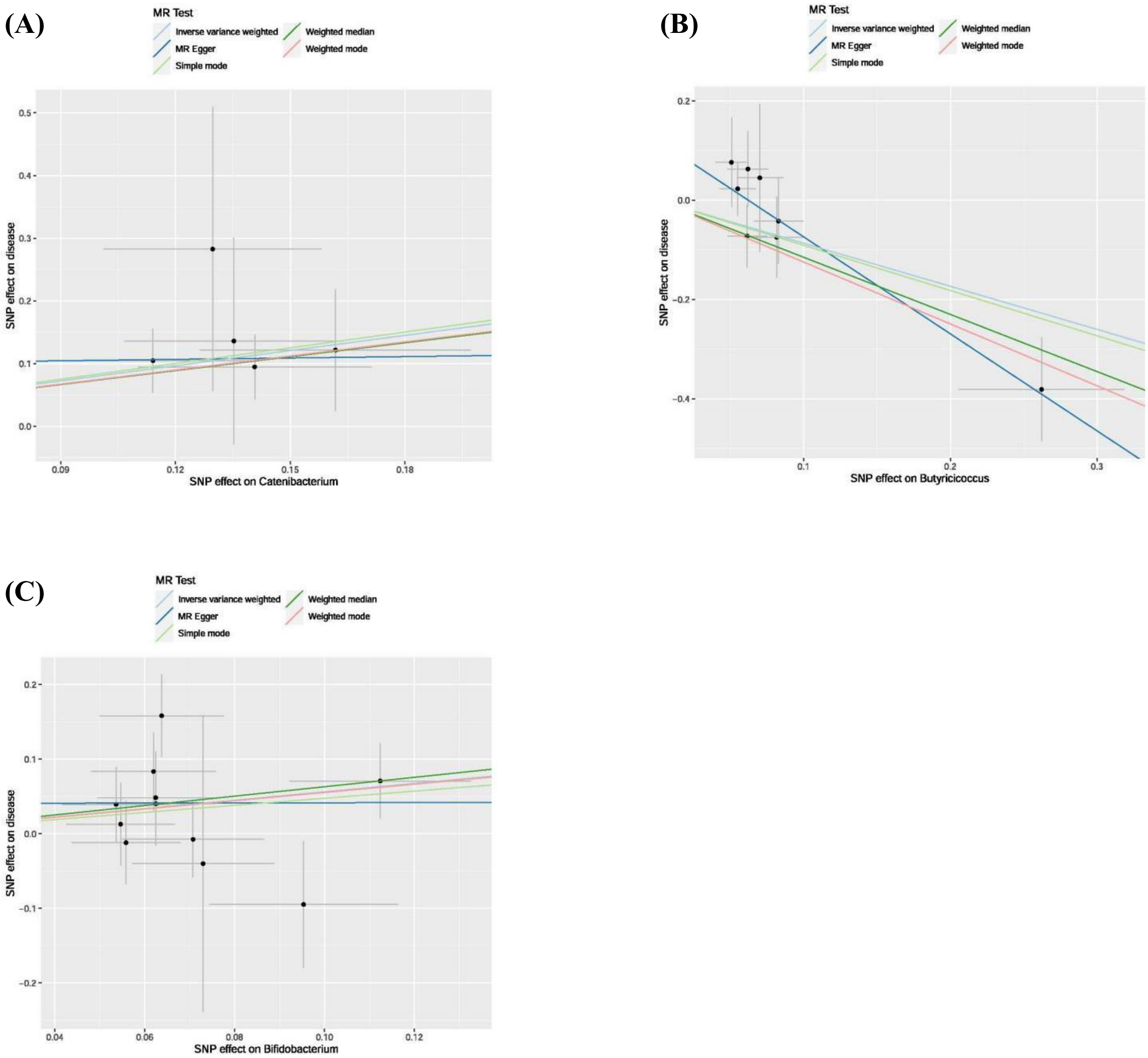

Figure S10. Scatter plots for the effect of Gut microbiota on PD.

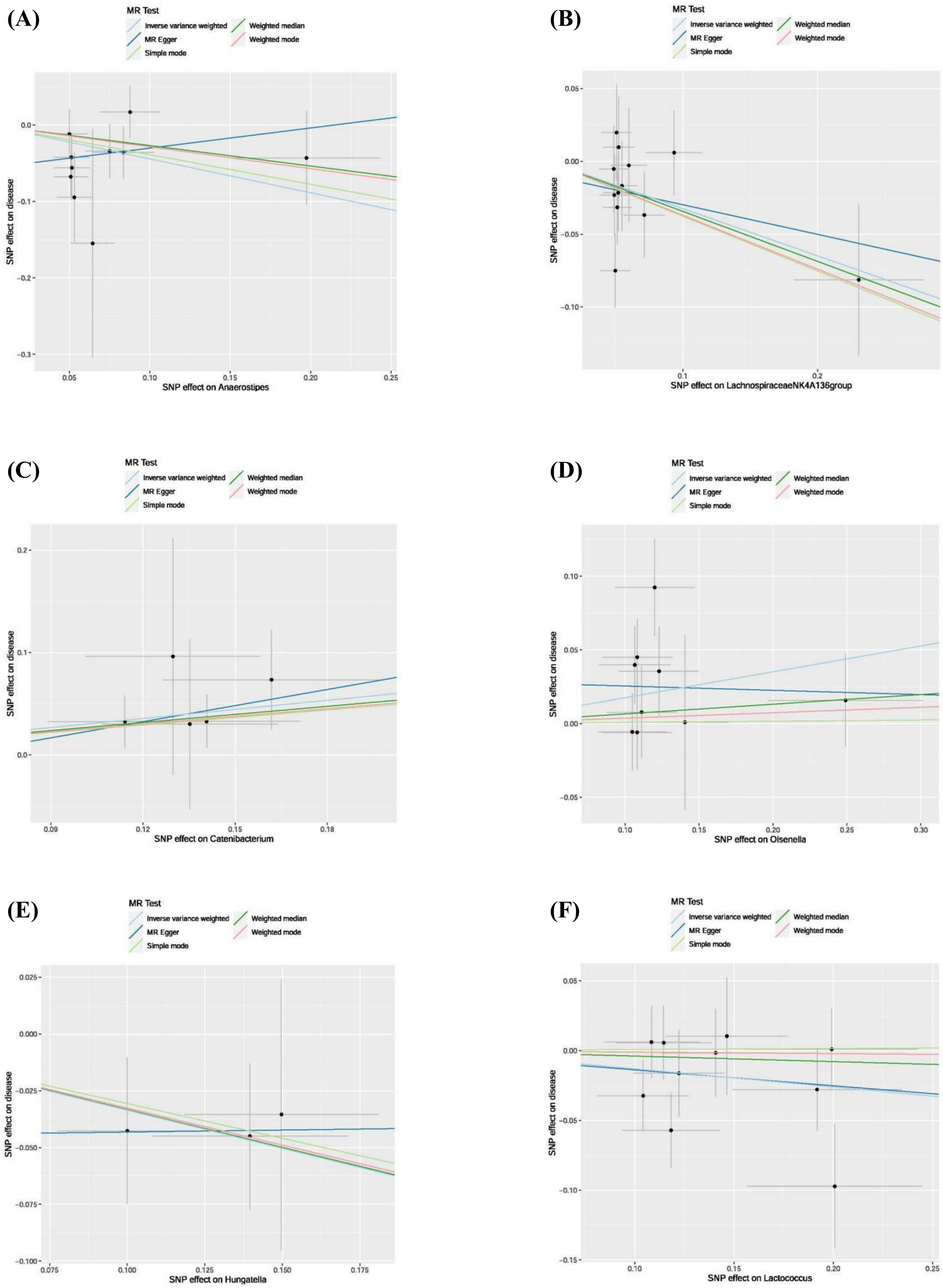

Figure S11. Scatter plots for the effect of Gut microbiota on PAD.

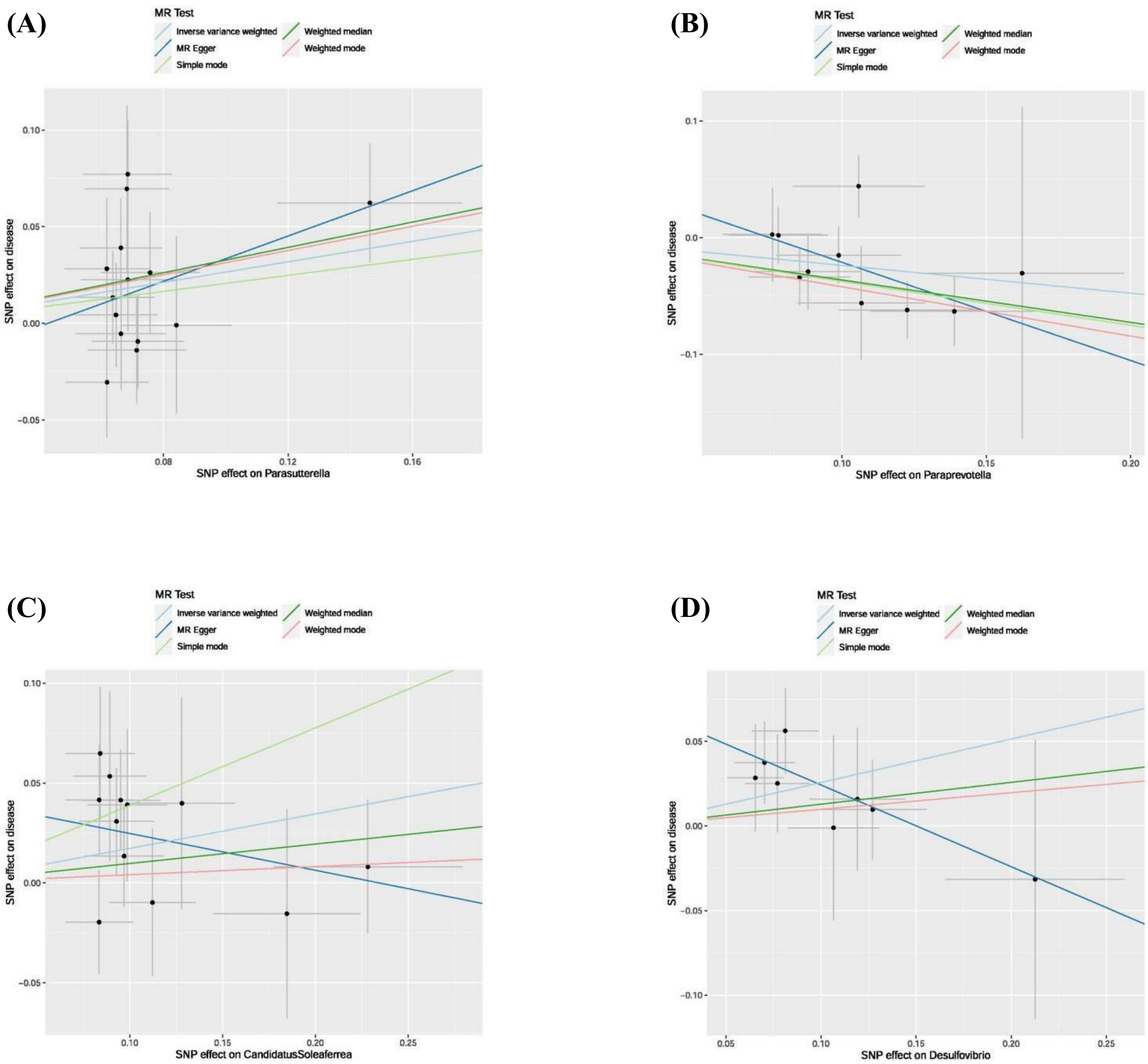

Figure S12. Scatter plots for the effect of Gut microbiota on CAD.

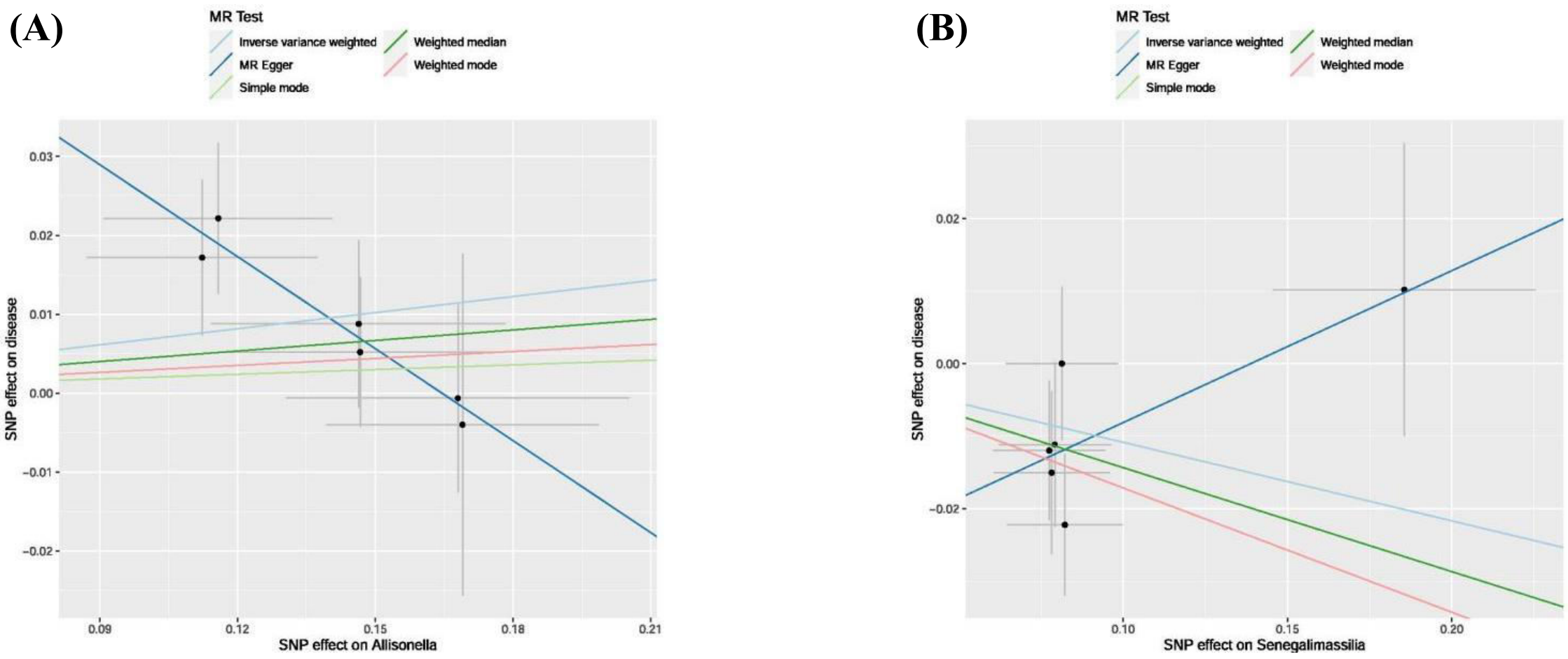

Figure S13. Forest plots for the effect of Gut microbiota on COPD.

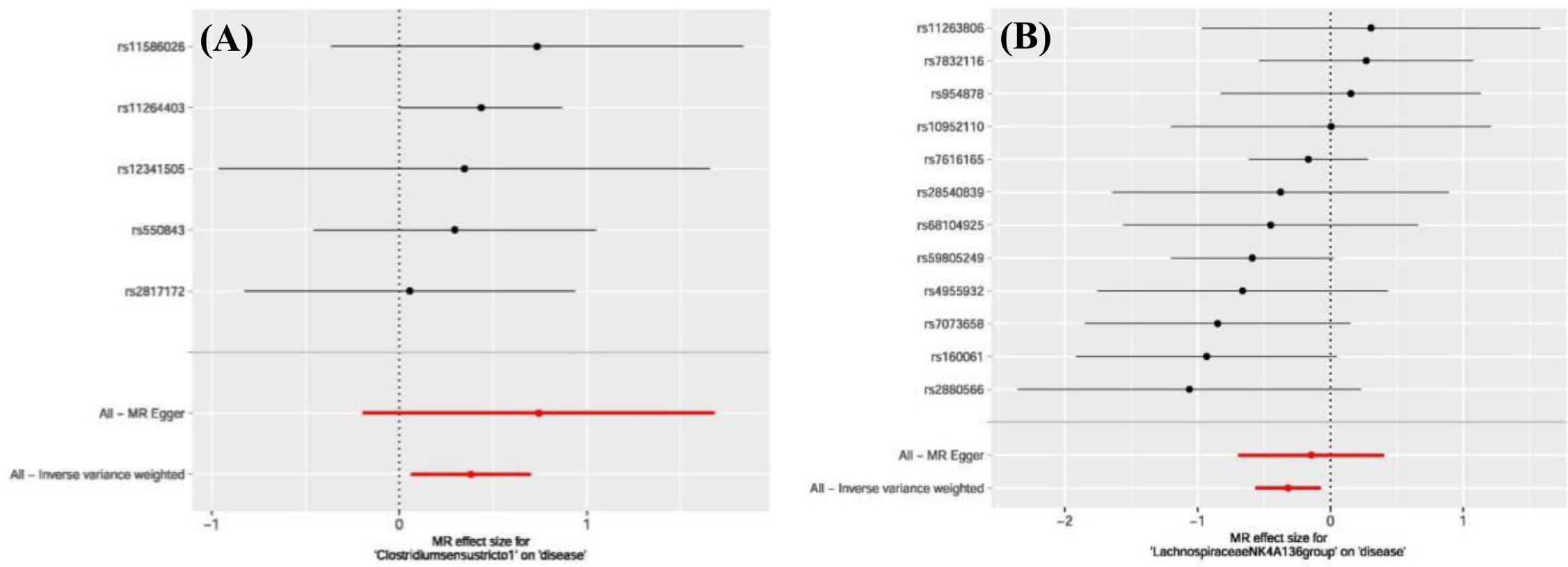

(A) MR effect size for "LachnospiraceaeNK4A136group" on "COPD"
(B) MR effect size for "Clostridiumsensustricto1" on "COPD"

Figure S14. Forest plots for the effect of Gut microbiota on GD.

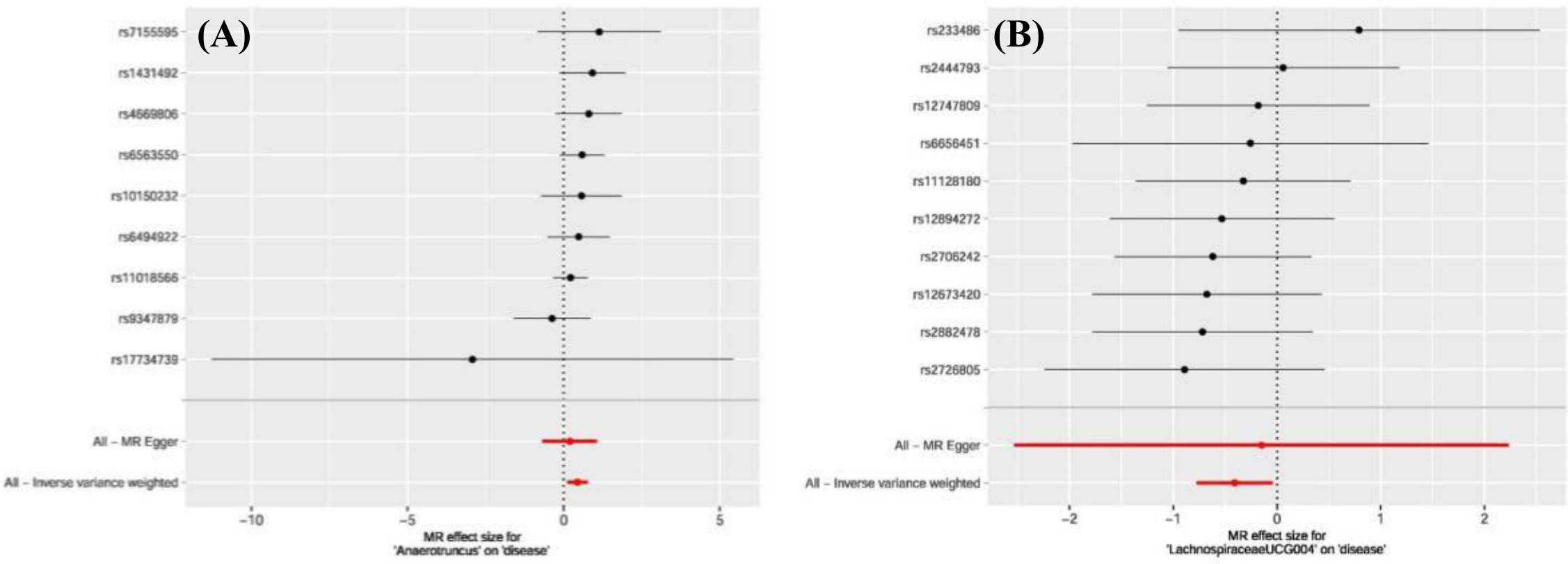

(A) MR effect size for "LachnospiraceaeUCG004" on "GD"
(B) MR effect size for "Anaerotruncus" on "GD"

Figure S15. Forest plots for the effect of Gut microbiota on ILD.

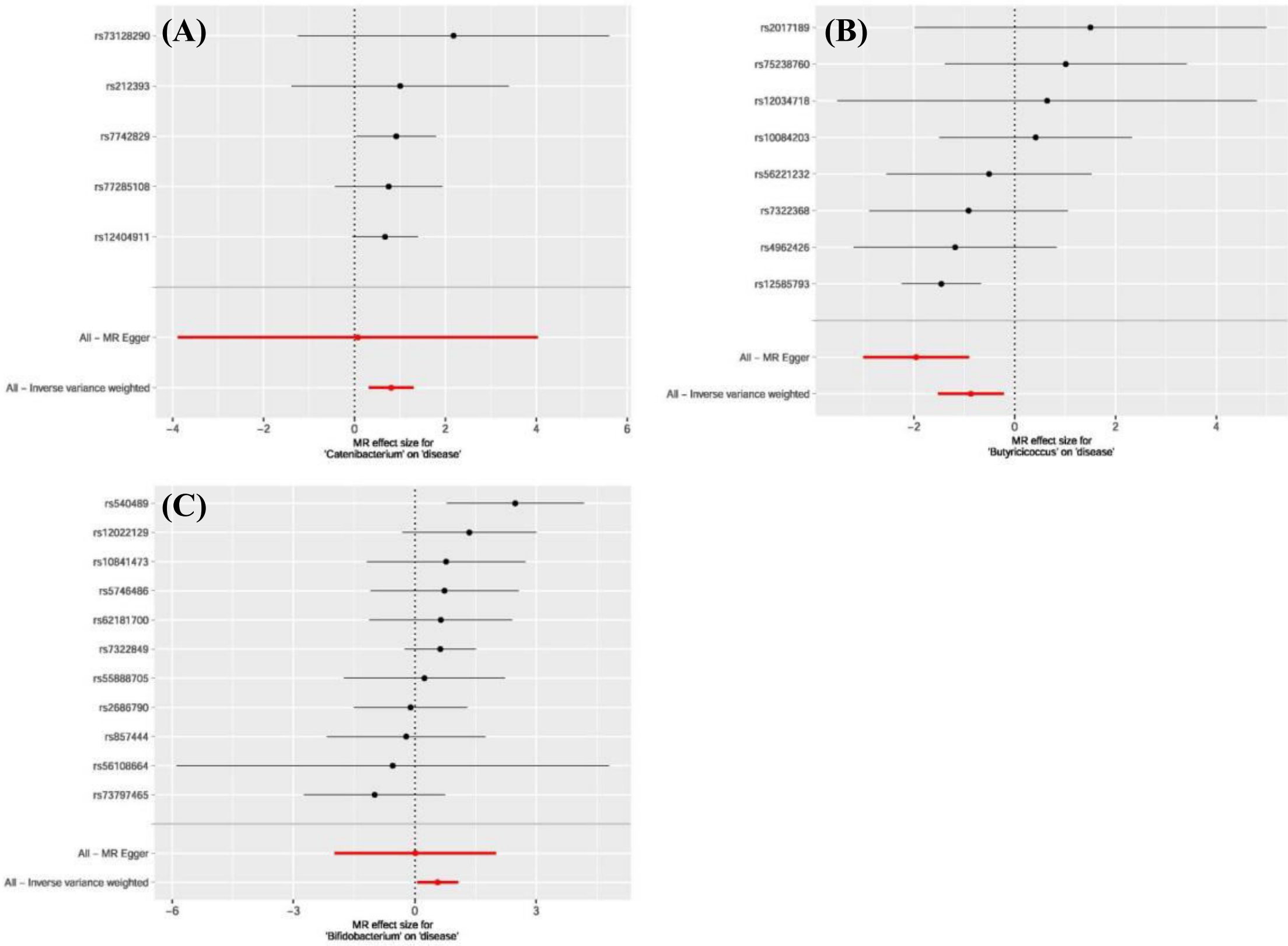

(A) MR effect size for "Catenibacterium" on "ILD"
(B) MR effect size for "Butyricicoccus" on "ILD"
(C) MR effect size for "Bifidobacterium" on "ILD"

Figure S16. Forest plots for the effect of Gut microbiota on PD.

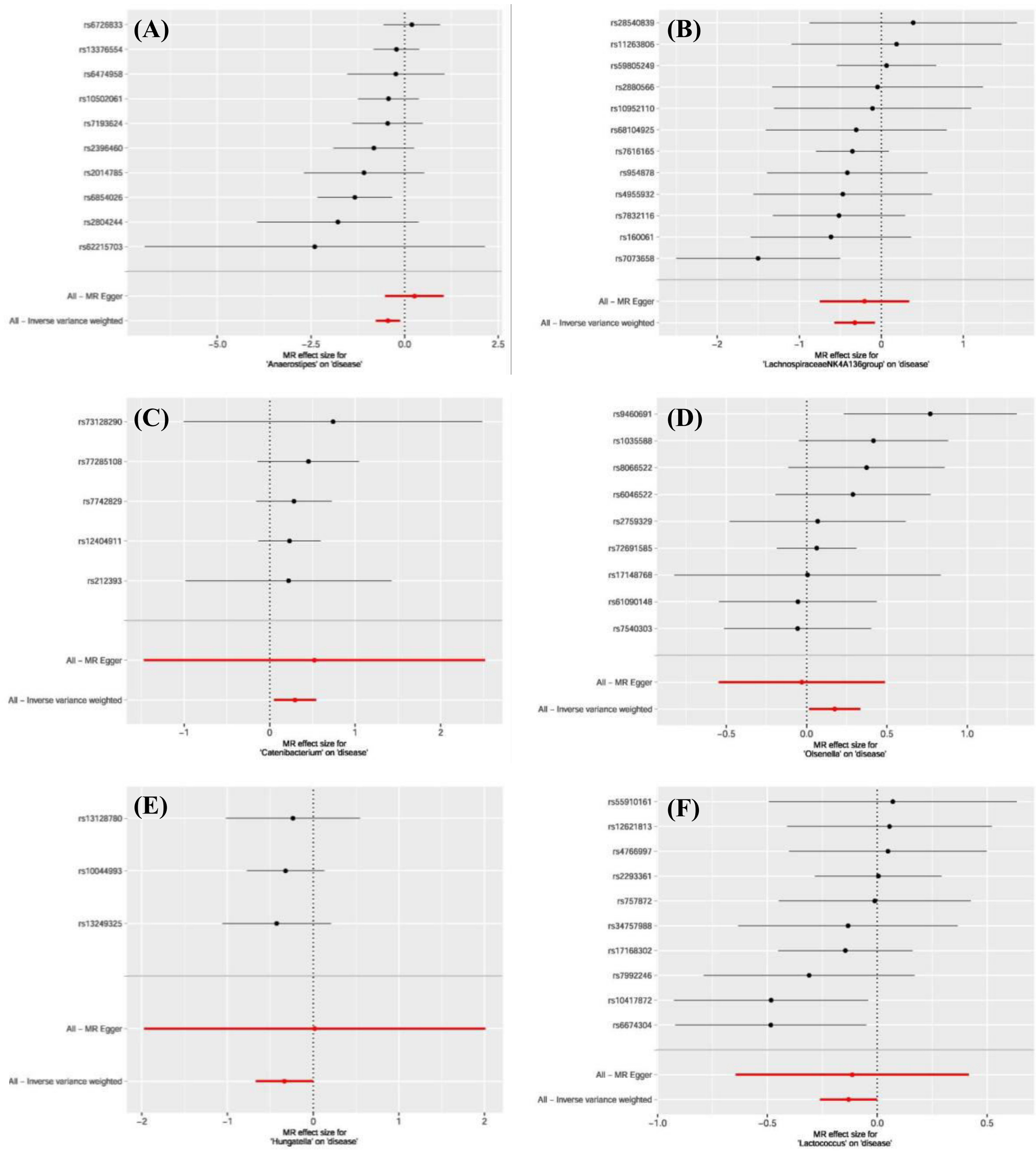

(A) MR effect size for "Catenibacterium" on "PD"

(B) MR effect size for "Olsenella" on "PD"

(C) MR effect size for "LachnospiraceaeNK4A136group" on "PD"

(D) MR effect size for "Hungatella" on "PD"

(E) MR effect size for "Anaerostipes" on "PD"

(F) MR effect size for "Lactococcus" on "PD"

Figure S17. Forest plots for the effect of Gut microbiota on PAD.

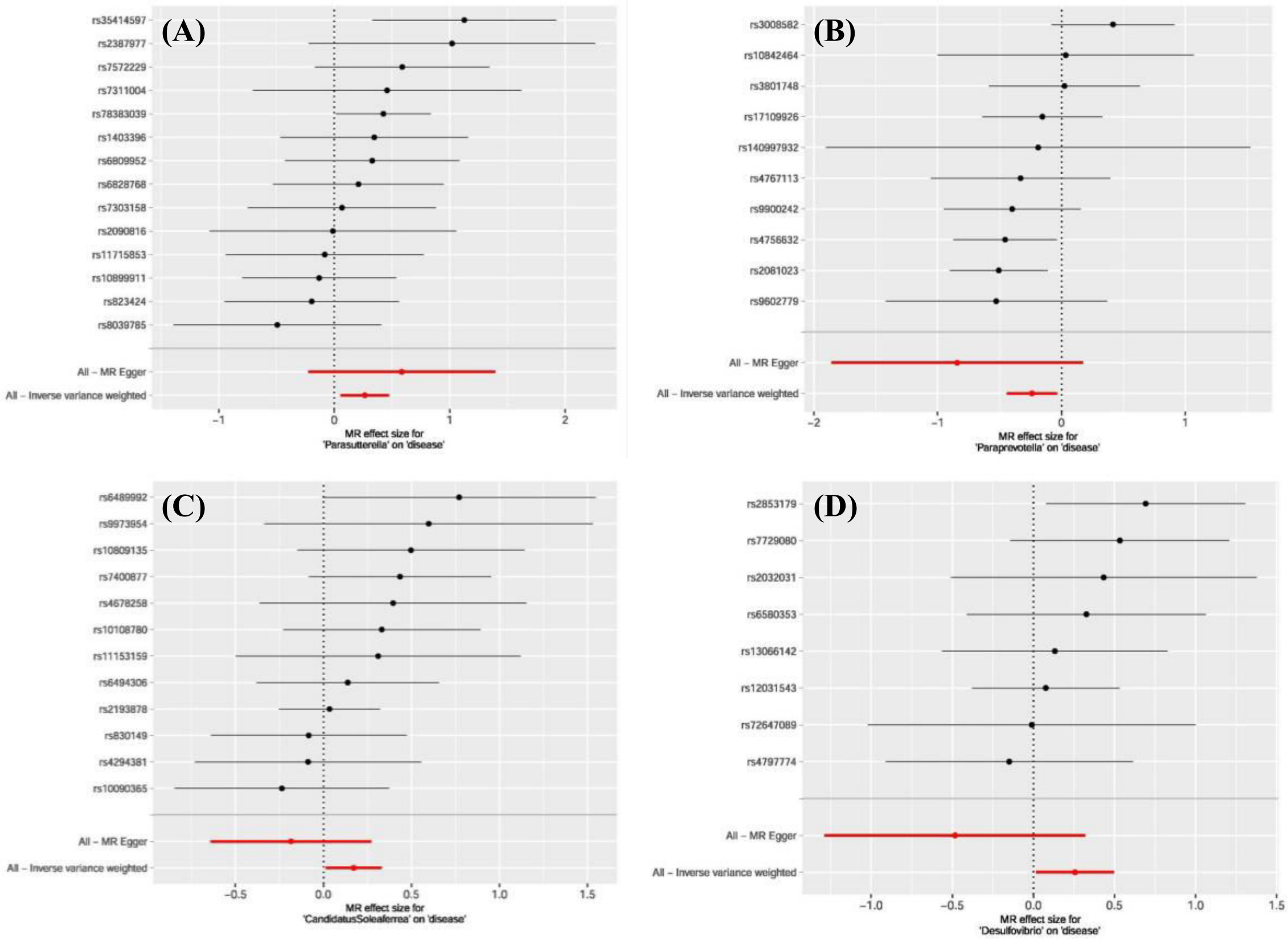

(A) MR effect size for "Desulfovibrio" on "PAD"

(B) MR effect size for "Paraprevotella" on "PAD"

(C) MR effect size for "CandidatusSoleaferrea" on "PAD"

(D) MR effect size for "Parasutterella" on "PAD"

Figure S18. Forest plots for the effect of Gut microbiota on CAD.

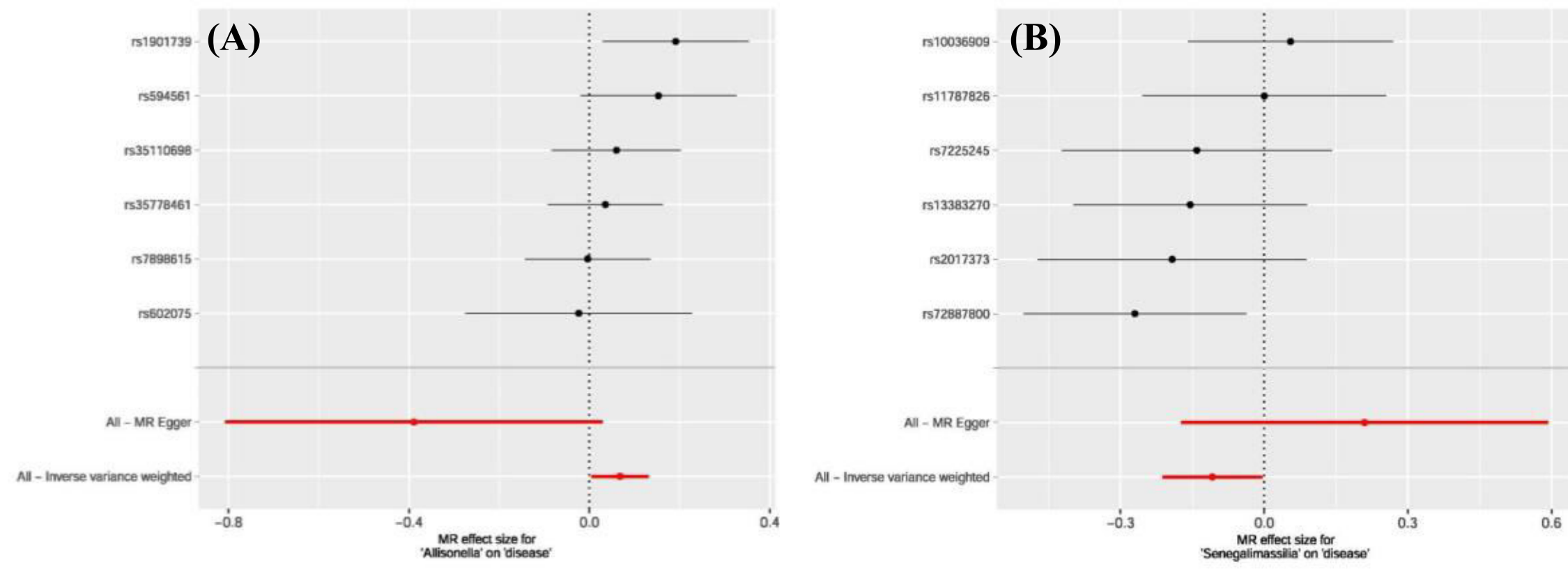

(A) MR effect size for "Senegalimassilia" on "CAD"

(B) MR effect size for "Allisonella" on "CAD"
